# Blue:Red LED Light Proportion Affects Vegetative Parameters, Pigment Content, and Oxidative Status of Einkorn (*Triticum monococcum* L. ssp. *monococcum*) Wheatgrass

**DOI:** 10.1021/acs.jafc.0c03851

**Published:** 2020-08-05

**Authors:** Maria Luce Bartucca, Marcello Guiducci, Beatrice Falcinelli, Daniele Del Buono, Paolo Benincasa

**Affiliations:** Dipartimento di Scienze Agrarie, Alimentari ed Ambientali, Università di Perugia, Borgo XX Giugno, 74, 06121 Perugia, ITALIA

**Keywords:** LED, wavelength, chlorophylls, carotenoids, hydrogen peroxide, malondialdehyde

## Abstract

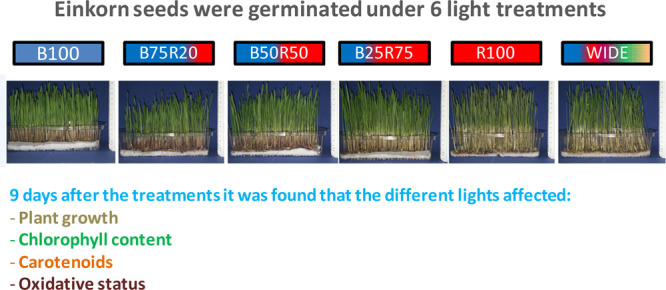

This work aimed to study the effect of some light spectra on the growth, oxidative state, and stress of einkorn wheatgrass (*Triticum monococcum* L. ssp. *monococcum*). To this end, six light treatments, having the same total incident photon flux density (PFD) of 200 μmol m^–2^ s^–1^, were applied to einkorn and compared: only blue light; only red; three blue:red combinations, at different proportions of total PFD (75:25%, 50:50%, and 25:75%, respectively); and a wide spectrum, taken as a control treatment, composed of blue (18% of PFD), red (18%), and intermediate wavelengths (64%). Light treatments affected the contents of pigments (chlorophylls and carotenes), hydrogen peroxide (H_2_O_2_), and malondialdehyde (MDA). These results revealed the changes in the oxidative status of wheatgrass, in response to the different light treatments. However, the dichromatic light with blue ≥50% of the total PFD appeared to be the best combination, guarantying good wheatgrass yield, increasing pigment content, and reducing H_2_O_2_ and MDA when compared to the other light treatments. Our findings also contribute to explaining the available literature on the effect of these kinds of light on the increase in phenolic compounds and antioxidant activity in einkorn wheatgrass.

## Introduction

Wheatgrass is currently recognized by scientific literature and consumers as an important source of many health-promoting compounds (e.g., phenolic compounds, carotenoids, etc.).^1,2^ In particular, the wheatgrass obtained from einkorn (*T. monococcum*L. ssp. *monococcum*) shows a high content of polyphenols, phenolic acids, and other antioxidants.^3−5^ Recently, Benincasa et al.^6^ demonstrated that the amount and composition of antioxidants in einkorn wheatgrass can be sharply affected by the light spectrum. In particular, the total polyphenol content can be increased by the blue radiation, and the total phenolic acid content by both the blue and red radiations, when compared to the white radiation used as the control treatment. The authors did not include the combinations of blue and red lights, but it is known that this may further increase the synthesis of certain compounds, differently from the monochromatic lights.^7^ In general, the use of specific light spectra, in place of the white light, is justified by the fact that sprout production is more and more carried out indoor with artificial light, both for the homemade and specialized production, and can be easily obtained by LED lamps, which have a long life span, low heat emission, and low power consumption.^8^

Blue and red lights are the major wavelengths perceived by plant photoreceptors (i.e., phototropins or cryptochromes for blue light and phytochromes for red light). The photoresponses are wavelength-dependent reactions,^9^ which take place with blue light in the region of 400–500 nm and with red light in that of 600–700 nm. Furthermore, it is noteworthy that blue and red lights can affect the plant morphology, physiology and development, photosynthesis, and primary and secondary metabolism (i.e., the synthesis of some phytochemicals).^7^ Although there is a large literature on the effect of blue:red light on the nutritional traits of plants, its role on the physiological, biochemical, and nutritional traits of wheatgrass, sprouts, and microgreens still remains unclear or unavailable for most plant species.^7,10^

Chlorophylls and carotenoids are key molecules operating in the photosynthetic pathway; the content of these pigments in plants is very responsive to light spectra and intensity to such an extent that their content can be increased or decreased by slight differences in the light.^11^ In general, it is not possible to depict a general trend of how species respond to different light spectra; there is a wide variability in the modulation of the plant pigments with the quality and quantity of the light.^12,13^ This evidence suggests that red and blue light should be investigated case by case, evaluating for each species the different sensitivity of its photoreceptors. On the other hand, variations in the content of these pigments with the light spectra should be carefully considered as they could indicate that the light treatments could also determine the insurgence of oxidative perturbations, affecting the cell, plant health status, as well as modifying the antioxidant activities.^14^ Particular attention should also be paid to the effect of light spectra on carotenoid contents for their pivotal role as light-harvesting pigments and scavengers of reactive oxygen species (ROS).^7^ Generally, when abiotic factors give rise to oxidative perturbations, an overproduction of ROS is observed. Among the ROS, increases of hydrogen peroxide (H_2_O_2_) can be recorded in response to different light spectra, as well as the accumulation of malondialdehyde (MDA). MDA is routinely used as an index of lipid peroxidation, as it is related to the oxidative damages to membranes, thus representing an indicator of the oxidative stress in plants.^14^

These premises show the intriguing perspective of studying the effects of blue:red LED lights on einkorn wheatgrass, with the aim to find suitable combinations capable of maximizing the content of pigments and minimizing that of oxidants. Therefore, some experiments were planned and carried out on einkorn wheatgrass grown with different light treatments, with the scope to evaluate the effect of the blue and red lights, alone or combined in different proportions, on chlorophyll *a* and *b*, and carotenoids, assessing whether the different light treatments caused oxidative perturbations, as revealed by the changes in H_2_O_2_ and MDA contents.

## Materials and Methods

### Plant Material and Sprouting

Einkorn grains (*T. monococcum* L. ssp. *monococcum*, cv. Monlis, TMoM) were incubated on a filter paper laid over sterile cotton contained in plastic trays (15 g of seeds per tray) and wetted with distilled water (150 mL) to guarantee constant water availability throughout the incubation period and prevent anoxia.^4^ The trays were placed in a growth chamber, in the dark, for 3 days after sowing (DAS) when most of the seeds germinated. Six different light treatments were then applied, all having the same total incident photon flux density (PFD) of 200 μmol m^–2^ s^–1^ (Table 1): only blue light (B100); only red light (R100); blue by 75% + red by 25% of total PFD (B75R25); blue by 50% + red by 50% of total PFD (B50R50); blue by 25% + red by 75% of total PFD (B25R75); and a wide spectrum (WIDE), composed of blue by 18%, red by 18%, and intermediate wavelengths by the remaining 64% of total PFD. In all the treatments, a light/dark photoperiod of 10/14 h was imposed. Three replicates per light treatment were performed. The light treatments were performed using the same LED lamps (DSA3 lamps) used by Tosti et al.^15^ The combination of wavelengths and the corresponding PFD of each light treatment are listed in Table 1. The growth chamber was maintained at 20 ± 1 °C and at a relative humidity of 70 ± 5%.

**Table 1:** Incident PFD for Each Radiation Wavelength in Each Light Treatment

	PFD (μmol m^–2^ s^–1^) of each wavelength^a^			
light treatment	blue	intermediate	red	total
B100	200	0	0	200
B75R25	150	0	50	200
B50R50	100	0	100	200
B25R75	50	0	150	200
R100	0	0	200	200
WIDE	36	128	36	200

a Blue: range from 400 to 500 nm, peak at 460 nm; red: range from 600 to 700 nm, peak at 660; intermediate: range from 500 to 600 nm, peak at 520 nm.

Wheatgrass was harvested at 9 DAS, collecting only the shoots. The sampled material was stored at −20 °C until analytical determinations, performed in triplicates. The fresh and oven-dried weights of shoots were measured on a subsample of 10 individuals per replicate.

### Photosynthetic Pigments

Einkorn seedlings were collected at 9 DAS, and the contents of chlorophyll *a*, chlorophyll *b*, and carotenoids were assessed. To this aim, the plant samples (1.5 g) were extracted, in a mortar and a pestle, with 85% acetone in water (v/v), adding small amounts of quartz sand to disrupt the tissues. The resulting suspensions were filtered, and the absorbance was determined spectrometrically at 452.5, 644 and 663 nm. The following equation was used to ascertain the content of the photosynthetic pigments:^16^
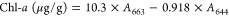

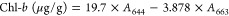

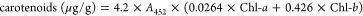


### H_2_O_2_ Assay

Plant tissues (0.5 g) were extracted, with a mortar and a pestle, in 4 mL of a buffer 50 mM KH_2_PO_4_/K_2_HPO_4_ (pH 6.5) and 1 mM hydroxylamine. Then, the extracts were centrifuged, and the H_2_O_2_ contents were assessed using a xylenol orange-based method.^17^ In detail, to 0.1 mL of the plant extract, 0.45 mL of a solution containing 200 μM (NH_4_)_2_Fe(SO_4_)_2_·6H_2_O and 50 mM H_2_SO_4_, and 0.45 mL of a solution containing 500 μM xylenol orange and 200 mM sorbitol, were added. These mixtures were then left to react for 30 min in the dark, and hydrogen peroxide was quantified spectrometrically at 560 nm according to Gay and Gebicki.^17^

### MDA Content

The level of lipid peroxidation was determined in einkorn seedlings, quantifying the plant concentration of MDA. To this scope, the seedlings (0.25 g) were homogenized in a solution containing 10% (w/v) trichloroacetic acid and 0.25% (w/v) thiobarbituric acid. The resulting suspensions were centrifuged for 15 min at 10,000*g*. Then, the supernatants were transferred into a water bath (95 °C) and warmed for 20 min. After quick cooling, the absorbance of the samples was determined spectrophotometrically at 532 and 600 nm, and the MDA content was calculated according to the study of Panfili et al.^18^

### Statistical Analysis

All data were analyzed by one-way ANOVA according to a randomized block design with three replicates. The average values of triplicate determinations ± standard errors are depicted. The means were compared by Fisher’s least significant difference (LSD) at *P* value < 0.05. The *R* statistical environment was used to perform the analysis.^19^

## Results

### Growth Parameters

**Figure 1:**
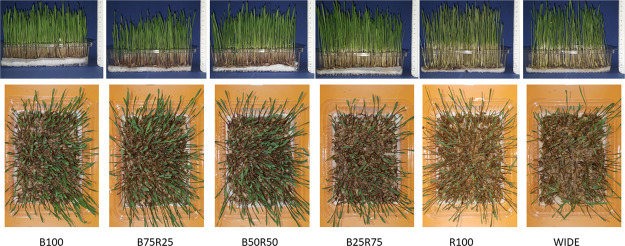
Side and top views of one tray of einkorn wheatgrass from each light treatment. B100: only blue light; R100: only red light; B75R25: blue 75% + red 25% of total PFD; B50R50: blue 50% + red 50% of total PFD; B25R75: blue 25% + red 75% of total PFD; WIDE = wide spectrum, composed of blue 18% + red 18% + intermediate wavelengths of the remaining 64% of total PFD.

**Figure 2:**
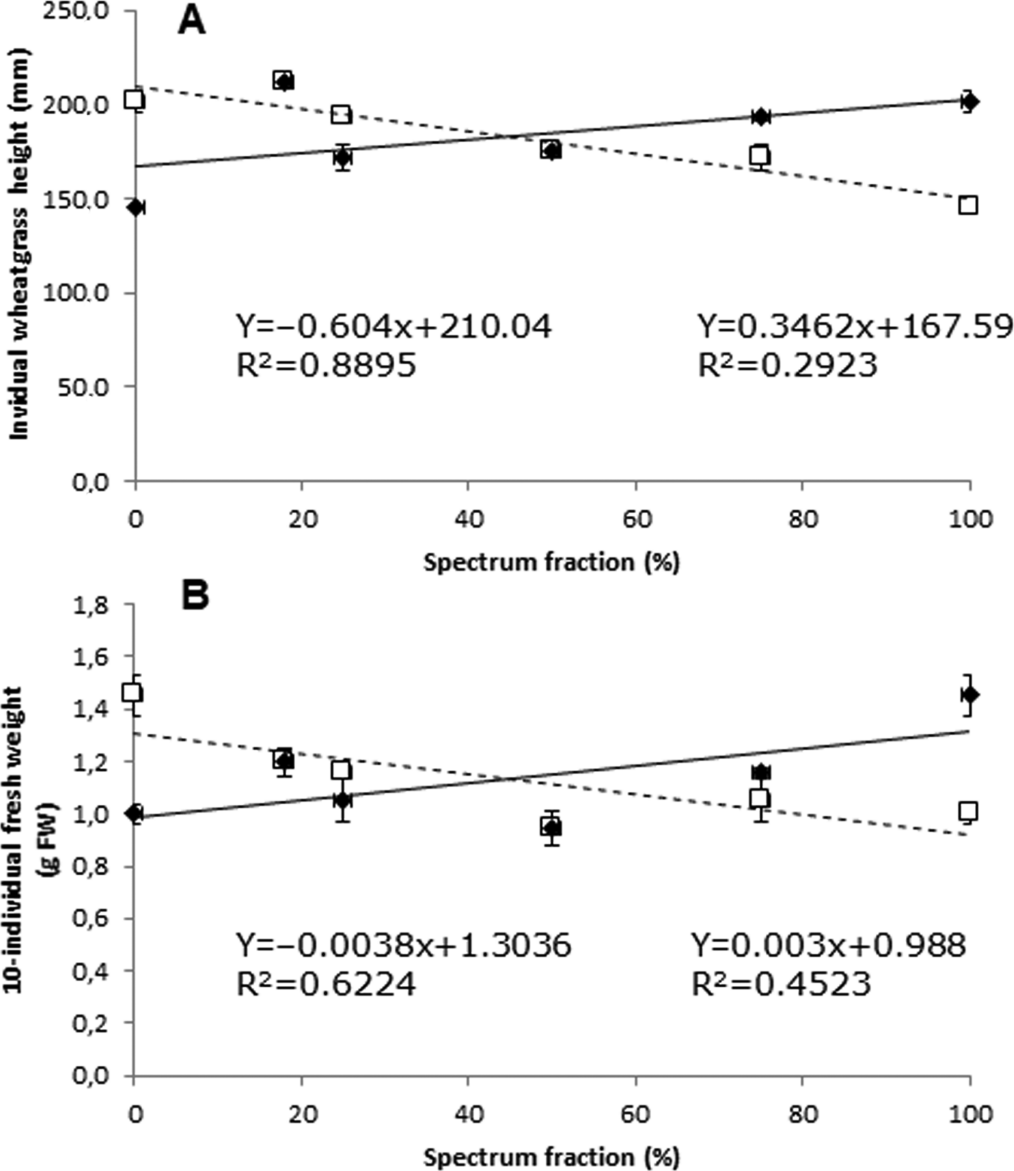
Relationships between either individual wheatgrass height (A) or fresh weight of 10 individuals (B) and percent fraction of total PFD for blue (white squares) and red (black diamonds) radiation in einkorn.

The growth parameters of the einkorn samples grown under the six different light treatments are reported in Table 2 and Figure 1, whereas Figure 2 well testifies the different vegetative statuses of einkorn wheatgrass at harvest. Both the average height and the fresh weight of 10 individuals of wheatgrass tended to increase with decreasing proportions of the blue radiation. The expansion of the leaves was greater and the green was the more intense the higher the percentage of the blue radiation. While the wheat grass grown only with the red radiation was thin, tall, and pale green. Einkorn wheatgrass grown under WIDE was the tallest, but the fresh weight was intermediate between that shown by the samples developed under blue- and red-only monochromatic lights. The dry matter concentration was not significantly affected by light treatments, with an average of 14.2%.

**Table 2:** Height (mm), Fresh Weight of 10 Individuals, and dry Matter Concentration (%) of Wheatgrass Grown with Different Light Treatments, all Having the Same Total Incident PFD of 200 μmol m^–2^ s^–1^^a^^,^^b^

light treatment	height (mm)	fresh weight (g) of 10 individuals	dry matter concentration (%)
B100	145 (±0.0)d	1.00 (±0.036)cd	14.2 (±0.17)
B75R25	172 (±7.3)c	1.05 (±0.C83)bcd	14.9 (±0.13)
B50R50	175 (±2.9)c	0.94 (±0.066)d	15.0 (±0.37)
B25R75	193 (±1.7)b	1.16 (±0.015)bc	14.2 (±0.45)
R100	202 (±6.0)ab	1.45 (±0.075)a	13.5 (±0.58)
WIDE	212 (±3.3)a	1.20 (±0.052)b	13.6 (±0.39)
F test			
significance	**	**	n.s.
LSD	1.3	0.182	1.17

a Standard errors in the brackets. LSD: least significance difference for *P* = 0.05; n.s.: not significant.

b B100: only blue light; R100: only red light; B75R25: blue 75% + red 25% of total PFD; B50R50: blue 50% + red 50% of total PFD; B25R75: blue 25% + red 75% of total PFD; WIDE = wide spectrum, composed of blue 18% + red 18% + intermediate wavelengths for the remaining 64% of total PFD.

### Pigments in Einkorn

**Figure 3:**
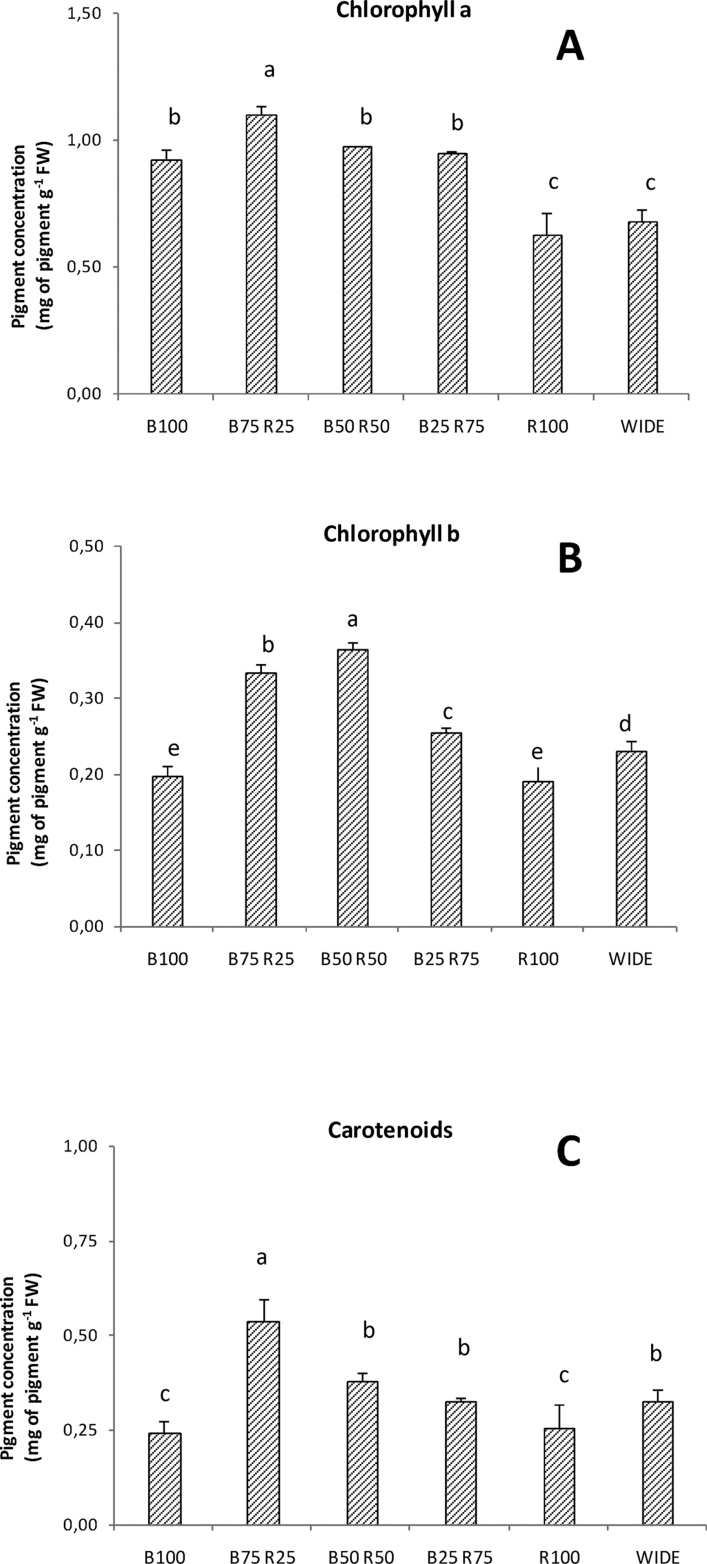
Chlorophyll *a* (A), chlorophyll *b* (B), and carotenoid (C) concentration (mg g^–1^ FW) found in einkorn wheatgrass grown with the different light treatments. Data are means + SD, and significant differences among samples are indicated by different letters (*P* < 0.05) (*n* = 3).

Figure 3a reports the contents of chlorophyll *a* found in the einkorn samples grown with the six different light treatments. Plants grown under B75R25 showed the highest pigment content, which was significantly different from all the other samples investigated, and reached the value of 1.10 mg g^–1^ FW. Plants grown with B50R50, B25R75, and B100 showed lower Chl-*a* contents of 0.97, 0.94, and 0.93 mg g^–1^ FW, respectively. These three values did not statistically differ among them. Einkorn wheatgrass grown under WIDE and R100 exhibited the lowest Chl-*a* contents, which were 0.68 and 0.63 mg g^–1^ FW, respectively.

Concerning Chl-*b*, a different trend was found in the light-treated einkorn (Figure 3b). Samples grown under B50R50 raised the content of this pigment at 0.36 mg g^–1^ FW, which was the highest value found in the samples treated with the six different lights. Plants grown under B75R25 showed a Chl-*b* content of 0.33 mg g^–1^ FW. Differently, the plants grown with the other light treatments showed a decreased Chl-*b* content. In particular, einkorn grown under B25R75, WIDE, B100, and R100 light treatments had pigment contents of 0.25, 0.23, 0.19, and 0.19 mg g^–1^ FW, respectively.

The content of carotenoids ascertained in the einkorn samples (Figure 3c), subjected to the six different light treatments, exhibited a trend more similar to that of Chl-*b* than Chl-*a*. However, the highest content of these pigments was found in the samples grown under B75R25 (0.53 mg g^–1^ FW). Wheatgrass grown under B50R50, B25R75, and WIDE light treatments showed the amounts of carotenoids of 0.37, 0.32, and 0.32 mg g^–1^ FW, respectively. These values did not statistically differ. The lowest content of pigments was found in the samples grown under B100 and R100, which showed the carotenoid contents of 0.24 and 0.26 mg g^–1^ FW, respectively.

### H_2_O_2_ Contents

**Figure 4:**
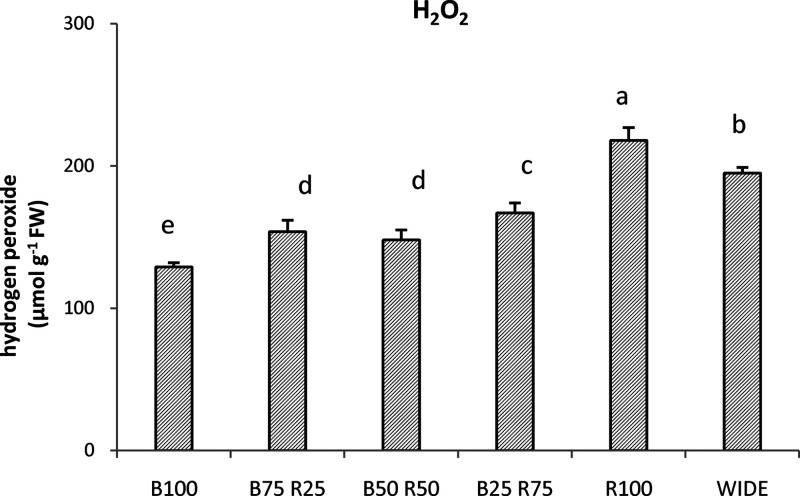
Hydrogen peroxide (H_2_O_2_) concentration (μmol g^–1^ FW) ascertained in einkorn wheatgrass grown with the different light treatments. Data are means + SD, and significant differences among samples are indicated by different letters (*P* < 0.05) (*n* = 3).

Hydrogen peroxide, a product very indicative of oxidative perturbation which can determine the stress to plants, was assessed in einkorn treated with the six different light combinations. Figure 4 shows the results of the H_2_O_2_ quantifications. The plants grown under R100 light treatment elevated the content of this oxidant to 218 μmol g^–1^ FW, representing the highest value following the different light treatments. The use of the WIDE light treatment showed a slightly lower H_2_O_2_ concentration, which, however, was significantly higher than that with the other remaining treatments. The content of H_2_O_2_ progressively decreased in einkorn treated with B25R75, B75R25, B50R50, and B100. The last treatment showed the lowest value (148 μmol g^–1^ FW).

### MDA Contents

**Figure 5:**
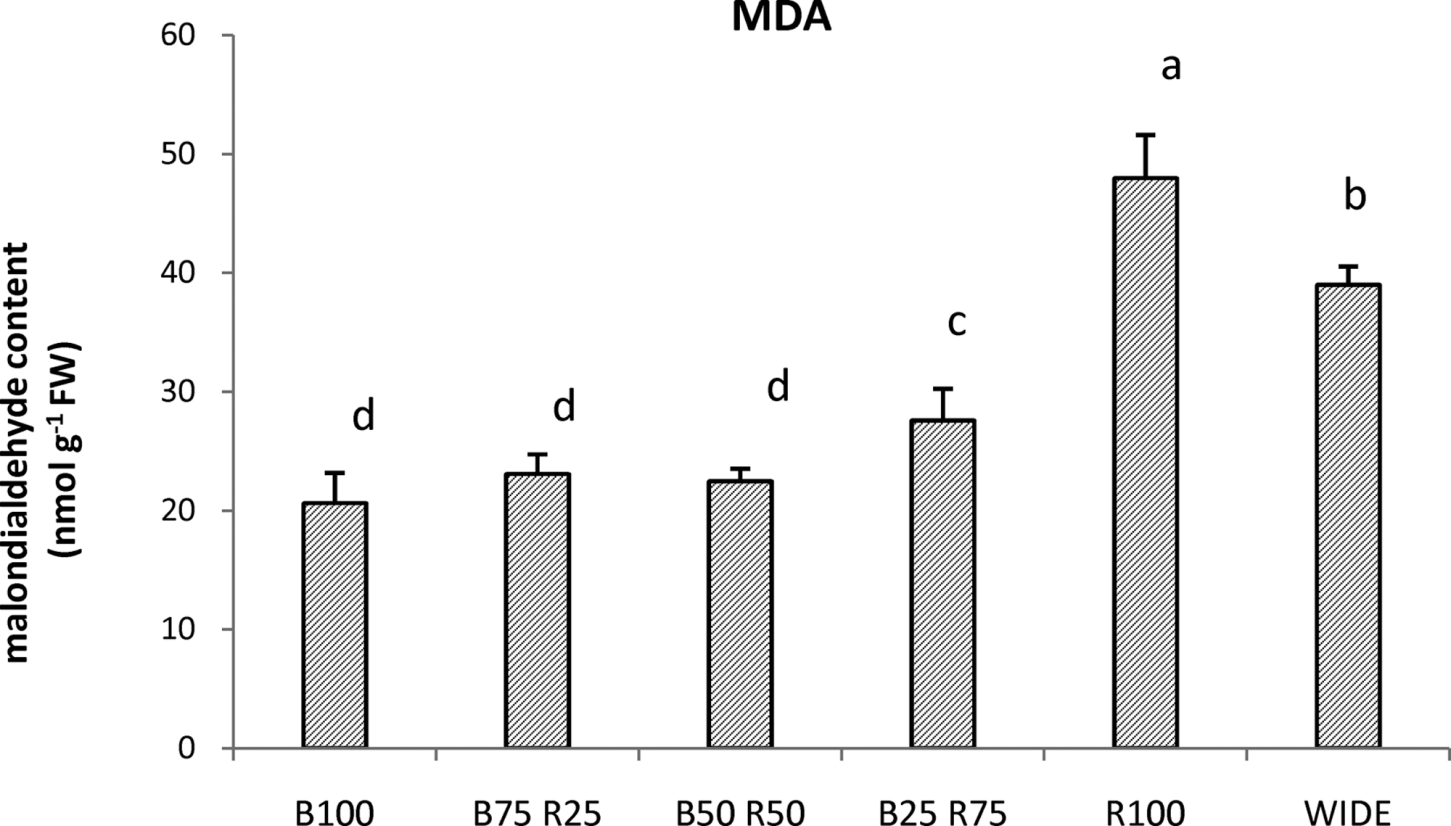
MDA content (nmol g^–1^ FW) found in einkorn wheatgrass grown with the different light treatments. Data are means + SD, and significant differences among samples are indicated by different letters (*P* < 0.05) (*n* = 3).

Figure 5 shows the data of the MDA content, assessed in the einkorn seedling subjected to the six different light treatments. The highest concentration of this product of lipid oxidation was found in samples treated with R100 (48 nmol g^–1^ FW). Plants grown under the other light treatments showed MDA values significantly lower than the plants treated with WIDE (39 nmol g^–1^ FW). In particular, plants treated with B25R75, B75R25, B50R50, and B100 showed MDA values of 28, 23, 22, and 21 nmol g^–1^ FW, respectively.

## Discussion

Plants adapt morphological and photosynthetic responses as a consequence of the light quality and quantity, and this is pivotal for their survival in a variety of dynamic environments.^20^ The same mechanisms are activated by crops subjected to the different light spectra, as obtained by LEDs,^21^ in a way that can be completely different among species with blue and red lights, and of their combinations. In our experiments, the differential effect of B100 and R100 on individual wheatgrass height and dry matter content was in line with the study of Benincasa et al.^6^ (Table 2), although the blue and red spectra of the two experiments were not exactly the same. Under the combinations of blue and red lights, wheatgrass appeared increasingly slim and pale green as the proportion of red light increased (Figure 1). A negative linear correlation was found between blue PFD and the fresh weight and height of wheatgrass. Such an effect was expected, based on the work by Hernández and Kubota,^22^ which demonstrated that the fresh shoot mass decreased by increasing the fraction of blue light. A possible explanation to this phenomenon was the decrease of the leaf area, associated with a reduction of the photosynthetic activity, because of the lowered plant capacity of intercepting the light. Pennisi et al.^10^ explained a similar trend, consisting in the reduction of basil yield under a higher fraction of blue light, as a consequence of a smaller leaf area and shortened internode length which worsen the light distribution within the canopy. However, it is worth to notice that, in our case, the dry matter accumulation (i.e., the product between the fresh weight and the dry matter concentration) was not affected; thus, the differences mainly concerned the tissue water status and the related cell expansion. Blue light is widely reported to reduce the cell wall extensibility and increase the cell turgor pressure and the rigidity of the hypocotyls.^23^ On the other hand, red light affects the stem elongation because of the phytochrome regulation.^24^ The effect on the seedling water status and fresh weight might also be the consequence of light treatments on stomatal functioning. Blue light has been reported to induce stomatal opening in a short-term exposure and to increase stomata number, as well as chloroplast functionality, in a long-term exposure.^23^ Similarly, red light is reported to stimulate the stomatal opening.^24^ Anyway, in the literature, the effect of blue and red light is not always univocal because of the differences in: (i) plant species; (ii) PFD values; and (iii) spectral composition (e.g., different R/B ratios or other wavelengths included).^23,24^ The slim and pale green appearance of wheatgrass obtained under the WIDE treatment was somehow not expected, with all the wavelengths being included. However, the blue and red portions of the total PFD were lesser than that in the typical white light, whereas intermediate wavelengths were more represented, and the overall light color tended to green.

At the whole plant level, different light spectra can decrease growth, condition the contents of photosynthetic pigments and antioxidants, and affect the nutritional status. These effects can be due to the plant’s capacity to perceive the differences in light quality through its photoreceptors, which can be active or inactive, with the composition of the light spectra in the range 300–800 nm.^25^ However, our experiments indicated a significant effect of the light treatments on chlorophyll *a*, *b*, and carotenoids (Figure 3). As a general trend, the monochromatic red and blue lights resulted in being less effective in stimulating the pigment contents, particularly those of chlorophyll *b* and carotenoids. In the case of dichromatic light treatments, depending on the relative blue:red ratio, the amount of pigments increased, and the best combination was found to be B75R25.

It is well known that chlorophylls *a* and *b* show strong absorption in the red (at 633 and 642 nm, respectively) and blue (430 and 453, respectively) regions.^26^ Plants modify their content of chlorophylls with the light spectrum. The controversial effect of red and blue light on the pigment contents is reported in the literature, indicating that plant responses are very different among species.^11^ In general, the monochromatic light alone (blue or red) has been shown to decrease the chlorophyll content in plants often.^11^ Furthermore, some authors reported for cucumber, spinach, radish, and lettuce, that, when blue light is present with other wavelengths, the chlorophyll content in the investigated species tends to increase with the given amount of blue light.^13,22,27,28^ Another interesting finding that emerged by comparing the relative content of chlorophylls *a* and *b* (Chl a/Chl b) is that passing from the blue to the red light this ratio significantly decreased (4.70 and 2.94 with B100 and R100, respectively). The monochromatic blue light tends, therefore, to actively stimulate the content of chlorophyll *a*, whereas the opposite effect was caused by the monochromatic red light, which, vice versa, positively affected the content of chlorophyll *b*. In general, the photosynthetic activity seems to proportionally increase with the amount of blue light present in the treatments.^13,22^ It is to be mentioned that the monochromatic lights alone, regardless of their wavelength, are unable to sustain an adequate photosynthetic process. However, some authors have evidenced the importance of the presence of blue light in the spectrum, as its absence can exert a substantial negative impact on the photosynthetic activity.^13,27,28^ Finally, the content of chlorophyll, if counted as the sum of chlorophylls *a* and *b*, further confirms that dichromatic light enhanced the chlorophyll content as higher as that with the proportion of blue. These effects can also be explained by the documented stimulatory action of the blue light, which can induce the relocation of chloroplasts which move to the cell surface with the scope to increase the photosynthetic efficiency.^29^ In this sense, chloroplasts showed a larger area in birch leaves (starch-free part of the chloroplast), and this effect was attributed to the blue fraction of the light. This reorganization of the chloroplasts prevented thylakoids from being too pressed against each other.^30^ Regarding the WIDE treatment, einkorn wheatgrass showed the chlorophyll content generally lower than that with dichromatic blue:red treatment and higher than those of samples grown with monochromatic lights. These differences reflected, as previously discussed, the spectral characteristics of the WIDE light, having a lower portion of red and blue (18% for each fraction), with the color tending to green, and further highlight the effectiveness of using specific spectra to increase the content of such pivotal molecules.

As far as the total carotenoids are concerned (Figure 3), their content was low with monochromatic light, whereas it increased with dichromatic light, particularly when the blue light was predominant with respect to the red light (B75R25). Carotenoids are photosensitizers and act as scavengers of ROS.^7^ As light-harvesting pigments, they collect light to pass the energy to the chlorophylls and protect them from higher energy forms.^7^ It is not possible to define a general trend among the amount of carotenoids produced by species and the treatment with monochromatic (red or blue light) and the dichromatic red and blue lights. The responses are species-specific; however, some studies have highlighted that blue light can induce the production of these pigments proportionally to their fractions in dichromatic treatments (red and blue). In particular, a positive correlation with the blue light was found in green leaves.^31,32^ The effectiveness of the combination of blue:red LED light in inducing the content of carotenoids was imputed to its capacity to regulate the carotenoid biosynthetic genes in Tartary buckwheat.^33^ Furthermore, it is to be mentioned that the content of carotenoids was lower in samples treated with WIDE light than in those treated with the dichromatic light B75R25, not significantly different from the other treatments with dichromatic lights, and higher than the monochromatic treatment with blue and red. This effect underlines one more time that the inductive effect of blue:red light on carotenoids, with a higher content of blue, deserves attention as other combinations of light could be ineffective in specifically stimulating a similar beneficial effect on wheatgrass.

Taking into account the effects exerted by the light treatments on the pigments, we investigated the content of hydrogen peroxide and MDA. This is with the scope to give evidence on the impact of light treatments on plants as these molecules can accumulate in response to oxidative perturbations. Many factors, for example, abiotic and biotic stresses, can give rise to the overproduction of H_2_O_2_, a harmful oxidant to cells for its capacity to progressively damage a series of molecules, even causing cellular death.^34^ This molecule is particularly reactive toward chlorophylls, proteins, lipids, DNA, and so forth.^34^ In our experimentation, the evaluation of the cellular amount of H_2_O_2_ produced by einkorn, following the different monochromatic or dichromatic light treatments, was functional to select the most suitable LED light treatment for the species, capable of avoiding oxidative perturbations and explaining the drop in pigments caused by some light treatments. The data from Figure 4 indicate that the blue light generally did not increase the amount of this oxidant, whereas the monochromatic red light was capable of inducing it. Consequently, the entity of damages caused by light treatments to cells was estimated by assessing the cellular MDA content. This molecule, a product of lipid peroxidation, is an important indicator of lipid degradation in response to various abiotic factors.^18^ The results of MDA evidenced, according to the amount of hydrogen peroxide found following the different light treatments, that the blue light, monochromatic or in combination with red light, did not cause oxidative perturbations to einkorn. In contrast, the treatment with red light alone raised the value of this lipid peroxidation product. Our findings on the pigment content, hydrogen peroxide, and MDA are in line with the results by Benincasa et al.,^6^ which demonstrated that a 2 day exposition of einkorn sprout to light was a too short period to record any relevant effect on antioxidant activities (expressed as DPPH and FRAP), whereas a week of exposition of this species to monochromatic LED lights modulated them. In particular, the stimulatory effect was ascertained in samples exposed to the blue light, whereas those grown with the red light showed decreasing antioxidant activities. The WIDE light, according to the findings reported here on pigments, was ascertained to be the second treatment most capable of increasing the H_2_O_2_ content and MDA accumulation. It can be reasonably postulated that a similar effect was the consequence of the low content of the blue fraction in the light composition, making the spectra ineffective in inducing antioxidant activity.

In conclusion, this work demonstrated that different proportions of blue and red lights could affect the pigment contents and the relative ratios and interfere with the oxidative status of einkorn wheatgrass. This fact is relevant and deserves attention as the ascertained reductions in the content of hydrogen peroxide and MDA, in einkorn wheatgrass, are the consequence of the increased content of some protective molecules, likely phenolic acids and other antioxidants, which are reported in the literature to be preferentially induced by the light spectra with a high proportion of blue light.

## Abbreviation

B25R75: light treatment with blue by 25% + red by 75% of total PFD
B50R50: light treatment with blue by 50% + red by 50% of total PFD
B75R25: light treatment with blue by 75% + red by 25% of total PFD
B100: light treatment with only blue light
Chl-*a*: chlorophyll *a*
Chl-*b*: chlorophyll *b*
DAS: days after sowing
DPPH: 2,2-diphenyl-1-picrylhydrazyl
FW: fresh weight
FRAP: ferric reducing antioxidant power
MDA: malondialdehyde
LED: light-emitting diode
LSD: least significant difference
PFD: photon flux density
R100: light treatment with only red light
ROS: reactive oxygen species
WIDE: light treatment composed of blue by 18%, red by 18%, and intermediate wavelengths by the remaining 64% of total PFD
